# Recombinant activated factor VIIa for the treatment of bleeding in major abdominal surgery including vascular and urological surgery: a review and meta-analysis of published data

**DOI:** 10.1186/cc6788

**Published:** 2008-02-15

**Authors:** Christian von Heymann, Sven Jonas, Claudia Spies, Klaus-Dieter Wernecke, Sabine Ziemer, Detlev Janssen, Jürgen Koscielny

**Affiliations:** 1 Department of Anesthesiology and Intensive Care Medicine, Charité-University Medicine Berlin, Campus Virchow-Klinikum and Campus Charité Mitte, Augustenburger Platz 1, 13353 Berlin, Germany; 2 Department of General and Transplantation Surgery, Charité-University Medicine Berlin, Campus Virchow-Klinikum, Augustenburger Platz 1, 13353 Berlin, Germany; 3 Sophisticated Statistical Analysis (SOSTANA) GmbH, Berlin, Wildensteiner Str. 27, 10318 Berlin, Germany; 4 Institute of Laboratory Medicine and Pathological Biochemistry, Charité-University Medicine Berlin, Charitéplatz 1, 10117 Berlin, Germany; 5 Med-i-Scene Concept GmbH, Schlesierstr. 9, 91085 Weisendorf, Germany; 6 Institute of Transfusion Medicine, Charité-University Medicine Berlin, Charitéplatz 1, 10117 Berlin, Germany

## Abstract

**Background:**

The purpose of this study was to determine the role of recombinant activated factor VII (rFVIIa) in abdominal, vascular, and urological surgery.

**Methods:**

We conducted meta-analyses of case series and placebo-controlled studies reporting on the treatment or prophylaxis of bleeding with rFVIIa regarding 'reduction or cessation of bleeding', 'mortality', and 'thromboembolism'.

**Results:**

All case reports (*n *= 15 case reports and 17 patients) documented an effect of rFVIIa in the treatment of bleeding. A meta-analysis of 10 case series revealed a reduction or cessation of bleeding in 39 out of 50 patients after administration of rFVIIa (estimated mean effect 73.2%, 95% confidence interval [CI] 51.0% to 95.4%) and a mean probability of survival of 53.0% (95% CI 26.4% to 79.7%). Among the rFVIIa responders, 19 out of 29 patients (66%) survived versus 1 out of 10 rFVIIa nonresponders (*P *= 0.003). Six out of 36 patients from the case series had a thromboembolic complication (estimated mean probability 16.5%, 95% CI 1.2% to 31.8%). Compared with a meta-analysis of eight placebo-controlled studies, no increased risk of thromboembolism was seen after administration of rFVIIa.

**Conclusion:**

The meta-analysis of case series showed that, in a mean of 73% patients, rFVIIa achieved at least a reduction of bleeding and that the probability of survival is increased in patients responding to rFVIIa. rFVIIa was not associated with an increased risk of thromboembolism compared with placebo.

## Introduction

Activated factor VII (FVIIa) plays a key role in hemostasis [1]. The effect of FVIIa is based first on its binding to tissue factor exposed on the subendothelium. The FVIIa-tissue factor complex is formed only in the region of endothelial damage, so that a local hemostasis occurs via the subsequent activation of factors IX and X and the formation of thrombin. After that, thrombin activates platelets and the factors V and VIII at the site of injury. Since 1996, a recombinant activated factor VII (rFVIIa) has been available (NovoSeven^®^; Novo Nordisk A/S, Bagsvaerd, Denmark). The rFVIIa doses used for registered indications (90 μg/kg body weight at intervals of 2 to 3 hours in hemophilia A or B with inhibitors, in acquired hemophilia, and in Glanzmann thrombasthenia and 15 to 30 μg/kg body weight every 4 to 6 hours for the treatment of bleeding in patients with congenital factor VII deficiency) exceed the physiological FVIIa concentration many times over. This results in an additional pharmacodynamic effect: irrespective of tissue factor as rFVIIa activates factor X directly on the surface of the platelets activated at the site of injury, so that large amounts of thrombin develop locally ('thrombin burst') and eventually induce the formation of fibrin from fibrinogen.

### Recombinant activated factor VII and prophylaxis of bleeding

While the registered indications are rather rare in abdominal and vascular surgery and in urology (hereinafter referred to as 'in the field of abdominal surgery') and are not meant to be further analyzed in this publication, some of the potential indications for rFVIIa listed in Table 1 are more frequent events. Basically, a distinction must be made between bleeding prophylaxis and the treatment of acute bleeding. Two placebo-controlled dose-finding studies are available on bleeding prophylaxis in patients without pre-existing coagulation disorders:

**Table 1:** Registered and potential indications of recombinant activated factor VII in patients undergoing abdominal surgery

Registered indications	Prophylaxis of bleeding related to surgical or invasive interventions as well as treatment of bleeding in patients with
	-Congenital hemophilia A or B if inhibitors are present (>5 Bethesda units) or if a strong increase of inhibitors must be expected upon administration of factor VIII or IX [41]
	- Acquired hemophilia [42]
	- Congenital factor VII deficiency [43]
	- Glanzmann thrombasthenia with antibodies to glycoprotein IIb/IIIa and/or human leucocyte antigen and presence or history of refractoriness to platelet concentrates [44]
Potential indications	Prophylaxis of surgical bleeding in patients with reduced activity or deficiency of coagulation factors, especially with specific inhibitors to plasmatic factors [45] and acquired von Willebrand disease [46]
	Treatment of bleedings after all conventional measures have failed in patients with
	- Chronic liver disease [47]
	- Thrombocytopathy [48]
	- Platelet-refractory thrombopenia [49]
	- Bleeding complications due to trauma or surgery in patients without any detectable systemic impairment of hemostasis (references in Tables 3 and 4)
	- Drug-induced bleeding, especially by hirudine (in connection with supportive measures), danaparoid, fondaparinux, and glycoprotein IIb/IIIa inhibitors [50]

During liver resection, a trend toward reduction of intraoperative blood loss was observed with the higher rFVIIa dose (80 μg/kg) (for example, 1,073 mL versus 1,422 mL on placebo; *P *= 0.07) [2]. However, the number of patients investigated (*n *= 63 in the placebo group and *n *= 59 in the 80 μg/kg rFVIIa group) was too small to achieve a significant result with the proposed reduction of blood loss of approximately 25%. A point of criticism in the design of this study was that rFVIIa was administered prior to the start of the operation, which led to an insufficient rFVIIa level during parenchyma dissection of the liver. In the second placebo-controlled study on the prophylaxis of bleeding, though not directly related to abdominal surgery, a significant reduction of the transfusion rate and perioperative blood loss was seen in retropubic prostatectomy as well as a reduction of the duration of operation in the study group treated with rFVIIa [3]. The small number of patients included in this trial (*n *= 36) subjects this study to the criticism of a lack of power. Furthermore, it should be noted that a median blood loss of 2,688 mL in the placebo group does not reflect average blood loss in retropubic prostate surgery. The overall study situation in the field of abdominal surgery in patients without pre-existing coagulation disorder does not constitute a sufficient basis to recommend rFVIIa for prophylaxis of severe bleeding.

### Recombinant activated factor VII and treatment of bleeding

Serious bleeding in abdominal surgical interventions occurs in approximately 2% to 5% of patients [4,5]. Bleeding complications can be caused or enhanced by coagulation disorders (Table 2). A marked acquired hemostatic disorder is often seen in massive transfusion (more than 10 units of packed red cells) with dilutional coagulopathy, hyperfibrinolysis, thrombocytopenia, hypothermia, and citrate excess with relative calcium deficiency [6]. In acute bleedings, rFVIIa constitutes an adjunct therapy to the replacement of platelets, fresh frozen plasma, and coagulation factor concentrates in patients with persistent uncontrollable bleeding after all conventional measures have failed, aiming at a rapid cessation of bleeding and thereby achieving (a) a reduction of further blood losses, (b) a reduction of further transfusion requirements, (c) prevention of hemorrhagic shock with subsequent multiple organ failure, and (d) improvement of baseline conditions for possible reoperation.

**Table 2:** Causes for peri- and postoperative bleeding complications and factors with influence on bleeding in abdominal surgery

Vascular lesion	Surgical	Intervention-related, accidental vascular lesion, suture insufficiency
	Congenital	Hereditary connective tissue diseases such as Ehler-Danlos syndrome, hereditary hemorrhagic telangiectases, cavernous giant hemangioma
	Acquired	Henoch-Schoenlein purpura, amyloidosis, gammopathies
Impairment of primary hemostasis (thrombocytic)	Congenital thrombocytopathy	Storage pool diseases (release disorders), Glanzmann thrombasthenia, Bernard-Soulier syndrome, Chediak-Higashi syndrome, Hermansky-Pudlak syndrome
	Congenital thrombocytopenia	Fanconi anemia, Wiskott-Aldrich syndrome, Thrombocytopenia-Absent-Radius syndrome
	Acquired thrombocytopathy	Treatment with platelet function inhibitors or nonsteroidal anti-inflammatory drugs, hypothermia, uremia, liver cirrhosis, extracorporal circulation, monoclonal gammopathies, malign thrombocytosis, volume replacement solutions, Dextran, high-molecular-weight HES solutions
	Acquired thrombocytopenia	Coagulopathy due to consumption or blood loss, extracorporal circulation, immunological, sepsis, drug-induced (for example, heparin-induced thrombocytopenia type II, but bleeding is rare)

Impairment of secondary hemostasis (plasmatic)	Congenital deficiency or reduced activity	Hemophilia A or B, rare deficiencies of other factors (fibrinogen, factors II, V, VII, X, and XI), factor XIII deficiency
	Acquired deficiency	Deficiency of vitamin-K-dependent factors during oral anticoagulation or liver disease, acquired hemophilia with inhibitors, coagulopathy due to consumption or blood loss
	Acquired reduction of activity	Hypothermia, acidosis, drug-induced: administration of unfractionated or low-molecular-weight heparin, of factor Xa inhibitors, of thrombin inhibitors, or of asparaginase. Diseases with impairment of fibrin polymerization (for example, acquired factor XIII deficiency) or volume replacement solutions (HES, gelatine)

Combined impairments of hemostasis (thrombocytic-plasmatic)	Congenital deficiency or reduced activity	von Willebrand disease
	Acquired deficiency	Organ-associated (for example, liver disease), acquired von Willebrand syndrome (for example, myelodysplastic syndrome), drug-induced (valproic acid), carriers of mechanic heart valves (aortic valve), aortic stenosis (high degree), high-molecular-weight HES solution

Impairment of fibrinolysis (hyperfibrinolysis)	Acquired	Hypothermia, acidosis, release of activators of fibrinolysis (for example, operations or damage of malignant tumors, uterus, prostate)

HES, hydroxyethyl starch.

In this paper, the case reports and case series published to date on the treatment of serious bleeding in the field of abdominal surgery are subjected to a meta-analysis regarding the success of bleeding control or a reduction of bleeding and regarding the correlation of this endpoint with mortality. Results are discussed keeping in mind that case series, and more likely case reports, are susceptible to publication bias. The risk of thromboembolic complications is analyzed systematically based on the case series with surgical patients and on placebo-controlled studies on the administration of rFVIIa in operated patients.

## Materials and methods

In a literature search, the databases Medline, Biosis, Embase, and Current Contents were screened by using key index terms such as rFVIIa, factor VIIa, recombinant activated factor VII, recombinant blood clotting factor 7a, recombinant FVIIa, or NovoSeven^® ^(period from 1980 to March 2006). The results were used to select clinical data and case reports on the clinical use of rFVIIa in abdominal surgery (no reviews or abstracts from conferences or scientific meetings). Moreover, the references listed in the literature found were looked through and the manufacturer was asked for publications on the subject matter described. All publications found with these key terms were analyzed as to whether they are about patients undergoing abdominal or vascular surgery, without pre-existing coagulation disorder, and treated with rFVIIa due to perioperative bleeding. Reports on trauma patients or patients with abdominal trauma were excluded. Moreover, all publications were analyzed for assessable information on the target variables 'cessation of bleeding or reduction of blood loss' ('reduction or cessation of bleeding'), 'mortality', and 'occurrence of thromboembolic complications'. These target variables were defined as 'met' (responder) or 'not met' (nonresponder) according to the judgment of the authors of the publications reviewed.

To differentiate case series from case reports, the former had to describe at least three patients being treated with rFVIIa for acute bleeding in one publication, at least one of them having to have undergone surgery in the abdominal region. The results of the descriptive analysis were reported in the form of means and ranges.

A subsequently performed meta-analysis of the case series investigated the mean relative frequency of the effects 'reduction or cessation of bleeding', 'mortality', and 'thromboembolism' of the patients treated with rFVIIa in a random effects model. Analysis was based on the assumption that the selected studies constitute a random selection, whose variance to be considered results from the addition of the individual study portion ${\hat{\sigma }}_{i}={\hat{p}}_{i}(1-{\hat{p}}_{i})/n_{i}\;$ and the estimated variance $\hat{\sigma }$ between the studies. A mean (weighted) effect along with its 95% confidence interval (CI) was calculated for each trial. In a second step, the placebo-controlled studies on the use of rFVIIa in surgical interventions published in the form of congress abstracts or original articles were evaluated with regard to the thromboembolic risk. Therefore, the mean (weighted) odds ratio of all studies was calculated along with the 95% CI, and the results were visualized using forest plots [7].

## Results

### Case reports and series

**Figure 1:**
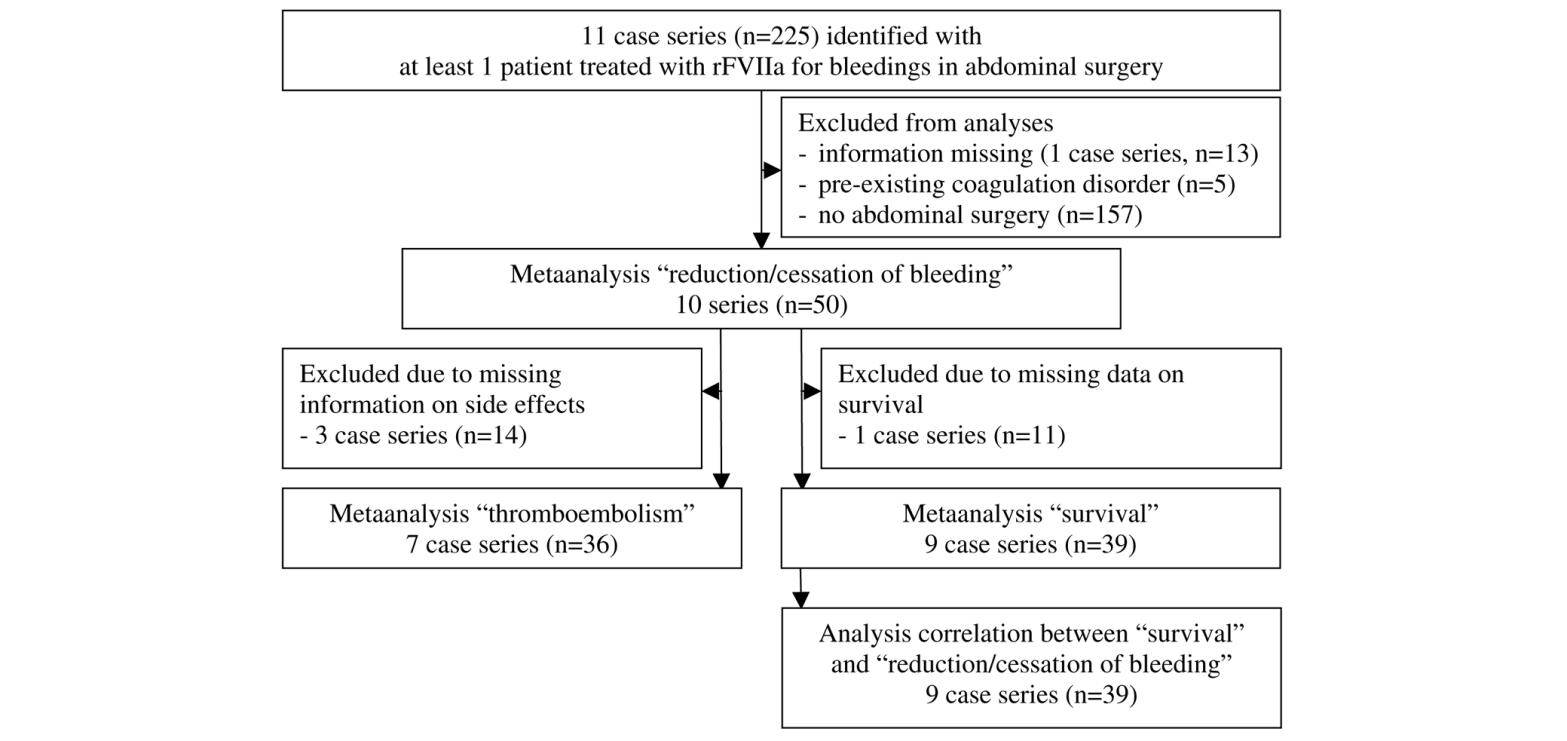
Flowchart on the analyses of case series. rFVIIa, recombinant activated factor VII.

There are no randomized studies on the treatment with rFVIIa for bleeding after and during interventions in the field of abdominal surgery. A literature search revealed 15 published case presentations reporting 17 patients (Table 3) and 11 case series (Figure 1). While all case reports met the inclusion criteria, one case series with 13 patients was excluded from analysis since the information given in that publication did not allow the operation procedures to be assigned to the individual cases [8]. In the other 10 case series, 50 out of 212 patients received rFVIIa in the context of an intervention in the abdominal region (Figure 1). The operations performed represent a wide range of major procedures in the field of abdominal surgery (Tables 3 and 4). The most frequent ones were operation of aneurysms of the aorta (*n *= 15) [9-12], pancreatic resection (*n *= 7) [11,13-17], colon resection (*n *= 8) [11,13,18,19], splenectomy (*n *= 5) [11,12,17], and urological operations (*n *= 8) [9,13,20-23]. Other operations/interventions were aortobifemoral bypass revisions (*n *= 1) [24], surgery of infected aortic prothesis (*n *= 1) [10], ileocolic anastomosis (*n *= 1) [13], operations for morbid obesity (*n *= 1) [13], liver hemangioma or liver rupture (*n *= 2) [13], ruptured venous malformation (*n *= 1) [25] and stomach cancer (*n *= 1) [15], laparotomy in the case of duodenal ulcer or small bowel resection (*n *= 3) [11,26,27], hematoma of the abdominal wall (*n *= 1) [28] or necrotizing enterocolitis (*n *= 4) [29], ischemic bowel resection (*n *= 1) [12], cholecystectomy (*n *= 3) [12,15,25], resection of sarcomas or teratomas (*n *= 2) [30,31], hematoma excision (*n *= 1) [32], endoscopic retrograde cholangiopancreaticography (ERCP) (*n *= 1) [13], and ERCP with sphincterectomy (*n *= 1) [33].

**Table 3:** Case reports on the treatment of severe bleeding with rFVIIa in patients undergoing abdominal surgery without known pre-existing coagulopathy

Reference	Age, gender, operation, other information	Indication for rFVIIa	rFVIIa dose	Bleeding after rFVIIa
White *et al*. [19] (1999)	1) 22 years, female, Crohn disease with colon resection due to bleeding	1) Persistent postoperative bleeding in spite of tranexamic acid and desmopressin	1) 2 × 90 μg/kg	1) Cessation
	2) 62 years, male, T-cell lymphoma with colon resection due to bleeding	2) Persistent bleeding in spite of relaparotomy	2) 2 × 90 μg/kg	2) Cessation, death due to multiple organ failure
Vlot *et al*. [27] (2000)	59 years, male, three laparotomies due to bleeding duodenal ulcer	Persistent bleeding in spite of surgical measures and tranexamic acid; rFVIIa in combination with octreotide	90 μg/kg every 2 hours over the span of 21 hours	Reduction
Chuansumrit *et al*. [28] (2002)	Premature infant, explorative laparotomy due to extraperitoneal hematoma of abdominal wall	Persistent bleeding in spite of FFP, cryoprecipitate and platelets	2 × 40 μg/kg	Cessation
Svartholm *et al*. [16] (2002)	50 years, female, pancreas necrosis and pseudocyst, pancreas resection, subtotal gastrectomy, splenectomy	Persistent bleeding from pancreas in spite of FFP, PCC, desmopressin, antithrombin, fibrinogen, tranexamic acid, and aprotinin	2 × 120 μg/kg (second dose after 5 hours)	Cessation after second dose
Danilos *et al*. [30] (2003)	45 years, female, resection of two big extraperitoneal sarcomas in the inguinal region	Life-threatening intraperitoneal bleeding with multiple bleeding sites in emergency laparotomy	80 μg/kg	Cessation 10 minutes after injection
Holcomb *et al*. [14] (2003)	45 years, male, necrotizing pancreatitis and explorative laparotomy with debridement of pancreas necrosis	Intraoperative bleeding, hypothermia, acidosis, coagulopathy, septic shock; massive transfusions during and after operation	120 μg/kg	Cessation
Schuster *et al*. [32] (2003)	55 years, male, hemorrhagic pancreatitis, compartment syndrome, excision of hematoma	Persistent bleeding	3 × 100 μg/kg	Cessation
Michalska-Krzanowska *et al*. [22] (2003)	1) 33 years, male, resection of the kidney	1) Persistent bleeding in spite of surgery/packing	1) 17 μg/kg	1) Cessation
	2) 56 years, male, prostatectomy	2) Massive, multifocal bleeding	2) 12 μg/kg	2) Cessation
Gielen-Wijffels *et al*. [21] (2004)	51 years, male, renal transplantation	Intra-abdominal bleeding after surgery, persistent hemodynamic instability in spite of reoperation	70 μg/kg	Stabilization of hemodynamics and hemoglobin value
Romero-Castro *et al*. [33] (2004)	53 years, male, endoscopic sphincterectomy	Persistent bleeding from the papilla with need for second endoscopy	4.8 mg	Cessation within 12 minutes
Dunkley and Mackie [20] (2003)	15 years, female, renal transplantation	Intraoperative, multiple bleedings, which cannot be controlled by conventional measures	135 μg/kg	Immediate reduction
Wordliczek *et al*. [17] (2003)	43 years, male, splenectomy and necrectomy in patient with acute pancreatitis	Persistent bleeding from drains	40 μg/kg; after 4 hours: 80 μg/kg	Reduction of bleedings from drains
Girisch *et al*. [31] (2004)	Premature infant, resection of sacrococcygeal teratoma	Persistent bleeding requiring emergency laparatomy	3 dosages, 150 μg/kg in total	Cessation
Sander *et al*. [23] (2004)	65 years, male, renal transplantation, thrombectomy	Massive intraoperative bleeding	30 μg/kg	Cessation
Raux *et al*. [24] (2005)	56 years, male, aortobifemoral bypass revision, pretreatment with aspirin and clopidogrel	Persistent bleeding in spite of FFP, platelets, fibrinogen, aprotinin as well as operations	90 μg/kg; after 2 hours: 45 μg/kg	Cessation; recurrence controlled with rFVIIa

FFP, fresh frozen plasma; PCC, prothrombin complex concentrates; rFVIIa, recombinant activated factor VII.

### Case reports and series: reduction or cessation of bleeding and mortality

An effect of rFVIIa was observed in all 17 cases published as case reports (Table 3). The 17 patients received an initial dose of 12 to 135 μg/kg body weight rFVIIa. Bleeding was stopped or, at the discretion of the author, reduced or stabilized with one rFVIIa administration in eight patients (Table 3) [14,20-23,30,33]. In three patients, a close temporal relationship between the administration of rFVIIa and control of bleeding was reported [20,30,33]. Six patients received two rFVIIa administrations [16,17,19,24,28] and three patients received three bolus doses [27,31,32], one of them receiving another seven bolus doses of rFVIIa after the drain losses declined until arterial embolization was performed [27] (Table 3).

In two populations of the case series included in the meta-analysis, there is no information on the dose in the subgroup of patients with interventions in the abdominal region [11,13]. In the other 30 patients, the initial rFVIIa doses were 30 to 40 μg/kg (*n *= 8), 80 to 120 μg/kg (*n *= 12) or 7.2 mg (*n *= 8), and 300 or 400 μg/kg (*n *= 2) (Table 4). The number of bolus doses administered was mentioned in seven case series for patients undergoing an operation in the abdominal region. No information on the number of bolus doses administered was found in three case series with a total of 24 patients [11,13,15]. Fourteen out of the 26 patients evaluable for the number of bolus doses received a single bolus (54%) [9,10,12,25,26,29], nine patients received two boluses (35%) [9,10,12,18,29], two patients three boluses (8%) [13], and one patient five boluses (4%) [12] (Table 4).

**Table 4:** Case series on the treatment of severe bleeding with rFVIIa in patients undergoing abdominal surgery without known pre-existing coagulopathy

Reference(s)	Total number of cases	Patients undergoing abdominal surgery without known pre-existing coagulopathy		rFVIIa dose	Reduction or cessation of bleeding after rFVIIa	Survivors
		Number	Indication for rFVIIa			
O'Connell *et al*. [11] (2003) and Laffan *et al*. [34] (2005)	40	11	Severe bleeding in patients undergoing colectomy (*n *= 4), operation of aortic aneurysm (*n *= 3), splenectomy (*n *= 2), intestinal resection (*n *= 1), resection of pseudocyst of pancreas (*n *= 1)	No information about subgroup	10/11	Not reported
Clark *et al*. [9] (2004)	10	4	Uncontrollable bleeding and after more than 15 packed red blood cells in elective operation of abdominal aortic aneurysm (*n *= 3) and after prostatectomy (*n *= 1)	1 × 7.2 mg (*n *= 3: 90, 101, and 97 μg/kg) 2 × 7.2 mg (90 μg/kg)	1/4	0/4
Mayo *et al*. [15] (2004)	13	4	Uncontrollable, life-threatening bleeding after operation of pancreas carcinoma (*n *= 2) or gastric carcinoma (*n *= 1) and cholecystectomy (*n *= 1)	Protocol: 7.2 mg (67.5 to 90 μg/kg), up to two more doses of 2.4 mg	3/4	3/4
Aggarwal *et al*. [18] (2004)	40	1	Uncontrollable bleeding in a patient with colectomy	2 × 90 μg/kg	0/1	0/1
Manning *et al*. [10] (2005)	8	6	Uncontrollable bleeding in vascular surgery interventions: aortic aneurysm (*n *= 5) and infected aortic prosthesis (*n *= 1)	1 × 40 μg/kg (*n *= 4) 2 × 40 μg/kg (*n *= 2)	5/6	4/6
Vilstrup *et al*. [26] (2006)	11	1	Intraoperative bleeding during operation of peptic duodenal ulcer	1 × 33.3 μg/kg	1/1	1/1
Haas *et al*. [25] (2005)	5	2	Uncontrollable bleeding in patients with ruptured venous malformation (*n *= 1) and after laparoscopic cholecystectomy (*n *= 1)	1) 1 × 120 μg/kg 2) 1 × 80 μg/kg	2/2	2/2
Filan *et al*. [29] (2005)	4	4	Uncontrollable liver bleeding in premature infants with laparotomy due to necrotizing enterocolitis (*n *= 4)	1) 2 × 100 μg/kg 2) 1 × 90 μg/kg 3) 2 × 300 μg/kg 4) 2 × 400 μg/kg	3/4	2/4
Biss *et al*. [12] (2006)	36	8	Uncontrollable bleeding in surgical patients with abdominal aortic aneurysm (*n *= 4), splenectomy (*n *= 2), ischemic bowel (*n *= 1), liver hematoma postcholecystectomy (*n *= 1)	3 × 30 μg/kg (*n *= 1) 1 × 90 μg/kg (*n *= 3) 2 × 90 μg/kg (*n *= 2) 3 × 90 μg/kg (*n *= 1) 5 × 90 μg/kg (*n *= 1)	5/8	2/8
Grounds *et al*. [13] (2006)	45	9	Intra- or postoperative bleeding in surgical patients with resection of sigma, liver hemangioma, liver rupture, prostatectomy, kidney transplantation, ileocolic anastomosis, endoscopic retrograde cholangiopancreaticography, morbid obesity, duodenopancreatectomy	No detailed information about subgroup	9/9	6/9

Total	212	50			39/50	20/39

Estimated mean effect					73.2% (51.0%–95.4%)	53.0% (26.4%–79.7%)

rFVIIa, recombinant activated factor VII

The meta-analysis of the case series on the outcome parameters yielded estimated mean effects (random effects model) of 73.2% (95% CI 51.0% to 95.4%) for reduction or cessation of bleeding and 53.0% for survival (95% CI 26.4% to 79.7%, one case series with 11 patients was not evaluable for this parameter). In the population of patients evaluable for survival, a reduction or cessation of bleeding occurred in 29 cases after administration of rFVIIa. In the remaining 10 patients, no reduction of blood losses occurred (Table 4) [9,10,12,15,18,29]. Only one out of 10 patients in which rFVIIa was not effective survived (10.0%) [15], whereas 19 out of 29 patients (65.5%) who responded to rFVIIa survived (*P *= 0.003) (*n *= 2 [15], *n *= 4 [10], *n *= 1 [26], *n *= 2 [25], *n *= 2 [29], *n *= 2 [12], and *n *= 6 [13]). Causes of death in spite of a reduction of blood loss after the administration of rFVIIa were multiple organ failure (*n *= 3 [12] and *n *= 1 [9]), persistent need of inotropic support [10], and persistent hypotension and acidosis [29] or no information was given on the cause of death (*n *= 3 [13] and *n *= 1 [15]).

### Case reports and series: thromboembolic complications

Thirteen case reports contain information on side effects [16,19-24,28,30,31,33], and two patients suffered a thromboembolic event after renal transplantation (partial thrombosis of the femoral vein and of the external iliac vein and common iliac vein [23]) and deep vein thrombosis at the site of a central venous catheter 1 month after rFVIIa [20].

Analysis of all case series yielded signs of a thromboembolic complication in six of 36 patients evaluable for this parameter. Besides rFVIIa, other procoagulants administered in the management of severe bleedings should be considered as a possible cause for these events: thrombosis of the iliac vein after colectomy [34], multiple small pulmonary embolisms after colectomy diagnosed by autopsy [34], arterial thrombus or transient digital ischemia in two children with arterial catheters [29], and thrombosis of the aortic graft and the renal artery after operation of a ruptured abdominal aortic aneurysm in one patient [12]. In one patient with hepatic rupture who developed a necrotizing colitis, a causal relationship with the administration of rFVIIa was considered to be possible by the authors [13].

In three case series (14 patients in total), there was no information on thromboembolic events [9,10,15] except the lack of signs of acute thrombosis mentioned for one patient with autopsy [10]. Based on the information from the remaining seven case series [11-13,18,25,26,29] with 36 patients, six of which experienced a thromboembolic event, the mean estimated probability of venous or arterial thromboembolisms was calculated to be 16.5% (95% CI 1.2% to 31.8%).

**Figure 2:**
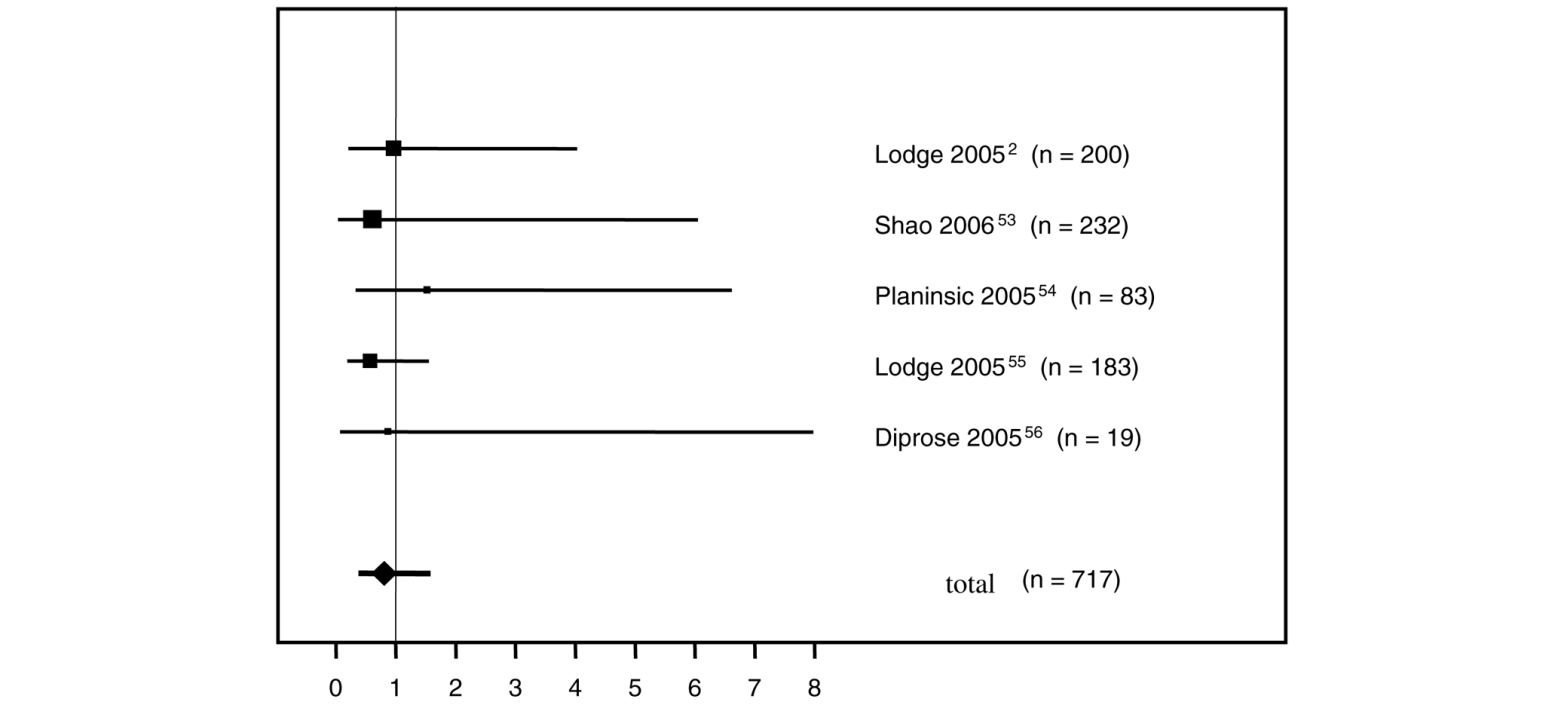
Forest plot of thromboembolic events in placebo-controlled studies on the prophylaxis of bleeding with recombinant activated factor VII in surgical interventions.

For the meta-analysis of placebo-controlled studies on the administration of rFVIIa in connection with surgical interventions, eight publications were identified, and in these a total of 285 patients received placebo and 555 patients received rFVIIa (Table 5). rFVIIa was administered for the prophylaxis of bleeding. The mean estimated risks of thromboembolism were 5.97% (95% CI 0.85% to 11.1%) for placebo and 6.42% (95% CI 1.08% to 11.75%) for rFVIIa, with an odds ratio for the entire population of 0.806 (95% CI 0.42 to 1.53) (Figure 2).

**Table 5:** Thromboembolic events in placebo-controlled studies for the prophylaxis of bleeding with rFVIIa in surgical interventions

Reference	Operation	Number of events/patients		Odds ratio (95% CI)
			
		Placebo	rFVIIa	
Levy *et al*. [51] (2006)	Dental operation	0/9	0/30	-
Raobaikady *et al*. [52] (2005)	Pelvic-acetabular surgery	0/24	0/24	-
Friederich *et al*. [3] (2003)	Prostatectomy	0/12	0/24	-
Lodge *et al*. [2] (2005)	Partial hepatectomy	3/68	6/132	0.97 (0.23–4.00)
Shao *et al*. [53] (2006)	Partial hepatectomy	1/81	3/151	0.62 (0.06–6.03)
Planinsic *et al*. [54] (2005)	Liver transplantation	3/19	8/64	1.53 (0.35–6.59)
Lodge *et al*. [55] (2005)	Liver transplantation	6/62	19/121	0.58 (0.22–1.52)
Diprose *et al*. [56] (2005)	Cardiopulmonary bypass	2/10	2/9	0.88 (0.10–7.95)
Sum		15/285	38/555	
Weighted sum		5.97 (0.85–11.1)	6.42 (1.08–11.75)	0.806 (0.42–1.53)

CI, confidence interval; rFVIIa, recombinant activated factor VII.

## Discussion

The meta-analysis of the present case series on the efficacy (reduction or cessation of bleeding) of rFVIIa in patients undergoing an operation in the field of abdominal surgery yielded an estimated mean effect of 73.2% of patients with a 95% CI of 51.0% to 95.4%. A number of case reports, though subject to a publication bias, document efficacy of rFVIIa by reporting a close temporal relationship between the administration of rFVIIa and cessation of bleeding [20,30,33]. These results confirm the experiences of different fields of operative medicine [35], describing the potential of treatment with rFVIIa for the control of serious bleeding in surgical patients.

The rate of cessation or reduction of bleedings after rFVIIa cannot be estimated by the analysis of case reports as these are associated with strong publication bias. Due to a lack of prospective trials, the analysis of case series may yield more reliable results. The mean survival rate of 53.0% determined from nine case series (95% CI 26.4% to 79.7%) demonstrates the poor prognosis of patients with severe postoperative bleeding that could not be controlled with conventional measures. The survival rate of 10.0% in nonresponders to rFVIIa was lower than in patients with reduction or cessation of bleeding after administration of rFVIIa (65.5%; *P *= 0.003). These results emphasize the relevance of sufficient control of bleeding for the outcome of patients. rFVIIa-refractory bleeding requires immediate measures of bleeding control (reoperation, packing, intraoperative interventional catheter procedures, and so on) to maintain the chance of survival.

Based on more than 10 years of experience with the treatment of patients with hemophilia, the frequency of side effects of rFVIIa can be expected to be less than one side effect per 1,000 standard doses [36]. As with any hemostatic drug, special attention should be given to thromboembolic events. Without a control group and due to the known increased thromboembolic risk of surgical procedures, it is not possible to establish an association with rFVIIa on the basis of the calculated mean rate of thromboembolism of 16.5%. Therefore, we performed an additional meta-analysis of placebo-controlled studies, in which the prophylactic use of rFVIIa was investigated in the context of surgical interventions. No significantly increased risk of thromboembolism was found in any of these studies. Our meta-analysis of studies using rFVIIa for therapeutic rather than prophylactic purposes did not show any significantly increased risk of thromboembolism after administration of rFVIIa compared with placebo. The mean estimated risk of thromboembolism of 6.42% in the eight studies on the prophylaxis of bleeding was within the 95% CI of 1.2% to 31.8% determined for the case series of bleeding therapy. Of note, thromboembolic complications were observed when rFVIIa was administered concomitantly with activated prothrombin complex concentrates (aPCCs) [36,37]. The thromboembolic risk due to the pretreatment with procoagulatory drugs should be taken into consideration in the benefit-risk assessment but *per se *does not constitute a contraindication for the administration of rFVIIa [35].

**Figure 3:**
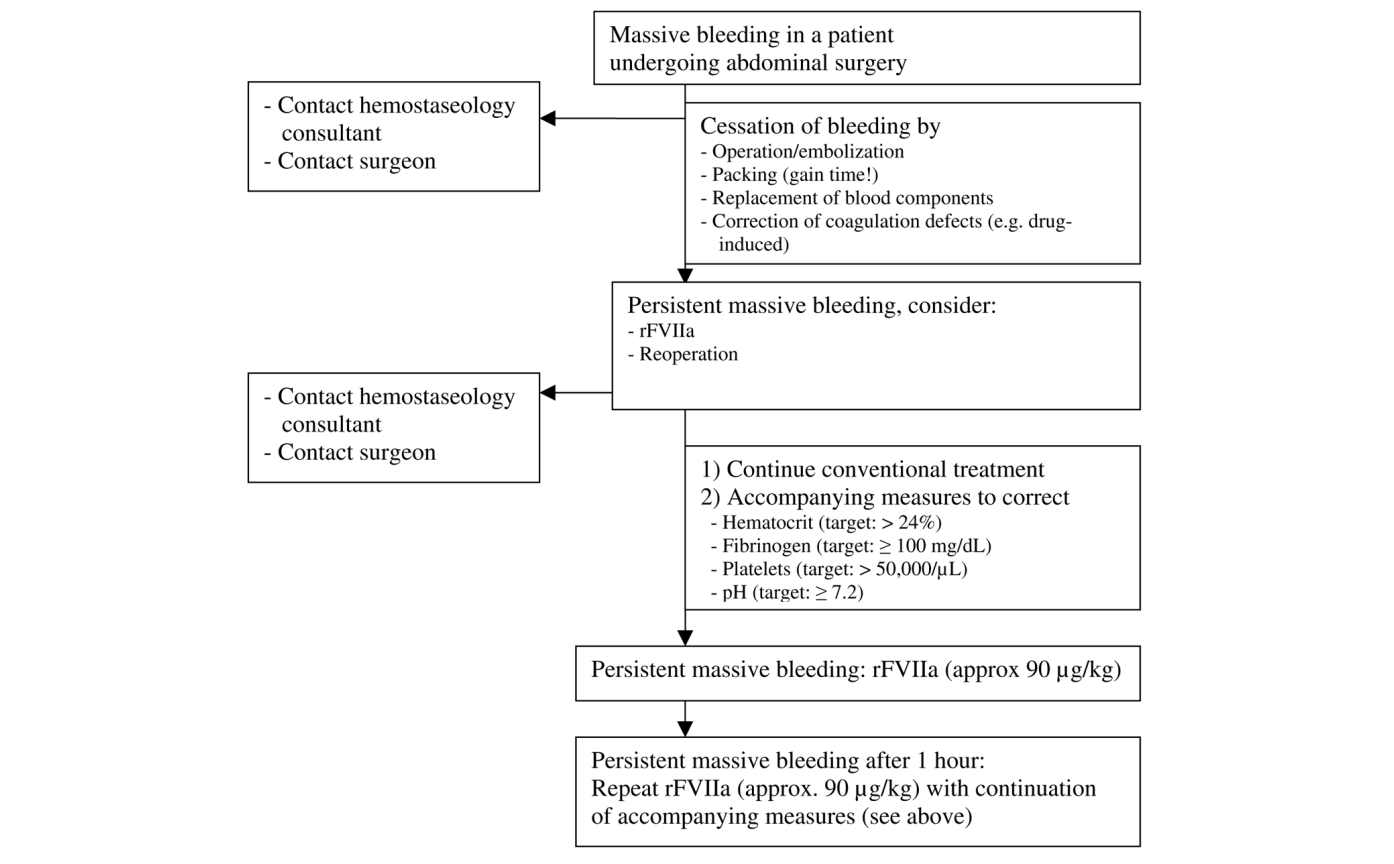
Diagnostics and measures for massive bleeding in abdominal surgery. rFVIIa, recombinant activated factor VII.

Since there are no controlled studies on bleeding therapy in abdominal surgery, the formal degree of evidence on the use of rFVIIa in patients with bleeding during and after operations in the field of abdominal surgery is low and currently corresponds to an expert opinion (degree of evidence C). To safely and effectively tailor the individual treatment of refractory bleeding with coagulation factor concentrates for which no evidence of efficacy can be derived from controlled studies, therapy algorithms considering the severity of bleeding, the patient's general health, and the availability of alternative hemostatic options, and so on can be helpful. Clark and colleagues [9] defined three criteria as a condition for the administration of rFVIIa: (a) transfusions of packed red cells corresponding to at least 1.5-fold the amount of blood volume (>15 units), (b) persistence of severe bleeding in spite of optimal conventional therapy, and (c) no foreseeable immediate surgical bleeding control. The treatment algorithm shown in Figure 3 describes a differentiated therapeutic and diagnostic procedure based on our own clinical experiences as well as on recently published recommendations [35]. It would be desirable to further substantiate this model and to provide evidence of the reduction of morbidity and mortality in randomized studies. The following findings are important when using the algorithm: (a) administration that is too late can compromise the success of treatment, (b) therapy with rFVIIa is useless in moribund patients in desolate overall circumstances, and (c) successful treatment of bleeding implies attempts for correction of acidosis (target pH of ≥7.2), fibrinogen (target of ≥100 mg/dL), platelets (target of ≥50,000/μL), and hematocrit (target of >24%) [35] which may also impact the efficacy of rFVIIa.

These findings are also supported by individual case series. The three nonresponders reported by Clark and colleagues [9] had already received between 25 and 44 packed red cell units at the time of rFVIIa administration and developed a serious coagulopathy. Mayo and colleagues [15] found that an improvement of the coagulation status had been achieved in patients responding to rFVIIa in the period between ordering of rFVIIa and administering of rFVIIa, whereas this was not the case in the group of nonresponders. Acidosis is associated with a reduced response to rFVIIa [38], so that its correction is of particular importance. The effect of rFVIIa is less impaired in patients with hypothermia [39]. Nevertheless, a body temperature in the normal range is of benefit to bleeding control [38,39].

Nowadays, with the budgeting in the health care system, the considerable costs of rFVIIa are an important factor in treatment decision. Therefore, pharmacoeconomic calculations are required which should involve costs of transfusion and intensive care treatment as well as length of stay in the hospital. Based on a population of patients who had received more than 5 units of packed red blood cells, a hypothetic model was able to demonstrate the cost-effectiveness of rFVIIa for administration after more than 14 units of packed red cells [40].

## Conclusion

The use of rFVIIa in serious bleeding during or after operations in the field of abdominal surgery has been described as effective and safe in various case reports and series. If there are no pre-existing coagulation disorders, there are currently no indications for the prophylactic administration of rFVIIa. However, rFVIIa can be taken into consideration as an additional therapeutic option if serious bleeding was refractory to conventional treatment. The degree of evidence about this corresponds to that of an expert opinion (degree C). According to the experience gained to date and to the placebo-controlled studies, safety is clinically sufficient, although an increased risk of thromboembolism for patients at risk is suspected. Prospective randomized studies need to investigate the efficacy and cost-effectiveness of rFVIIa in this indication in order to allow a final assessment of the importance of this treatment to be made.

## Key messages

• While all case reports documented recombinant activated factor VII (rFVIIa) to be effective, which represents a substantial publication bias, the meta-analysis of the case series showed a reduction of bleeding in 73.2% of patients.

• Patients who responded to the administration of rFVIIa with reduction or cessation of bleeding had a significantly higher probability of survival.

• Thromboembolic complications occurred in six of 36 patients from the case series (16.5%), which was not increased compared with the meta-analysis of thromboembolic events from the eight placebo-controlled studies.

## Abbreviations

CI = confidence interval; ERCP = endoscopic retrograde cholangiopancreaticography; FVIIa = activated factor VII; rFVIIa = recombinant activated factor VII.

## Competing interests

CvH, SJ, SZ, and JK have received speaker's honoraria from pharmaceutical companies, including Novo Nordisk Pharma GmbH (Mainz, Germany), which are engaged in the field of hemostasis. Furthermore, CvH, SJ, and CS declare that they took part in multicenter studies sponsored by Novo Nordisk Pharma GmbH. DJ declares that his company received honoraria from Novo Nordisk Pharma GmbH for activities as an independent medical advisor, including collaboration in the drafting of this manuscript. K-DW declares that he has no competing interests.

## Authors' contributions

CvH contributed to the conception and design of the work, sampling of data, analysis and interpretation of the data, and drafting of the article. SJ and CS contributed to the conception and design of the work, sampling of data, interpretation of the data, and revision of the article for content. SZ contributed to the conception and design of the work, interpretation of the data, and revision of the article for content. K-DW contributed to the analysis and interpretation of the data, revision of the article for content, and approval of the version to be published. DJ contributed to the conception and design of the work, sampling of data, interpretation of the data, and drafting of the article. JK contributed to the conception of the work, interpretation of the data, revision of the article for content, and approval of the version to be published. All authors read and approved the final manuscript.
